# Perceived barriers and facilitators of physical activity among Saudi Arabian females living in the East Midlands

**DOI:** 10.1016/j.jtumed.2021.11.002

**Published:** 2021-12-14

**Authors:** Abdullah Almaqhawi

**Affiliations:** a Department of Orthopaedics, Trauma and Sports Medicine, School of Medicine, University of Nottingham, United Kingdom; b Department of Family and Community Medicine, College of Medicine, King Faisal University, Al Ahsa, KSA

**Keywords:** الدافع, المعوقات والميسرات, النشاط البدني, السعودية, النساء, النشاط الرياضي, نمط الحياة., Barriers and facilitators, Females, KSA, Lifestyle, Motivation, Physical activity

## Abstract

**Objective:**

This study was conducted to explore the potential barriers to and facilitators of physical activity in Saudi Arabian females living in the East Midlands region of the United Kingdom.

**Methods:**

A qualitative study was conducted with Saudi Arabian females currently living in the East Midlands, along with the completion of International Physical Activity Questionnaires (IPAQs). The Semi-structured interviews were digitally recorded, transcribed, and analysed using a narrative thematic approach; IPAQ data were descriptively analysed. A total of 12 interviews were conducted. Five refined themes and 19 sub-themes were identified.

**Results:**

The results of this research showed that attractive climate, availability of open spaces, easy gymnasium accessibility, low cost, independent student lifestyles, food habits, efficient transportation, and UK culture serve as facilitators, while reduced availability of individual women-only gyms, non-engaging fitness instructors and reduced attention to different age groups serve as barriers to physical activity for Saudi Arabian females in the East Midlands.

**Conclusions:**

Although the results concerned Saudi Arabian females in the East Midlands, the participants' perspectives showed that more appropriate action could be taken to raise awareness of the advantages of being active among females in general. Drawing on a service delivery model based on the stages of change and including relevant stakeholders in developing and implementing effective interventions, strategies and policies must be developed to promote the participation of Saudi Arabian females and female expats from other countries in physical activity in the UK.

## الملخص

**أهداف البحث:**

أجريت هذه الدراسة لاستكشاف العوائق والميسرات المحتملة للنشاط البدني لدى النساء السعوديات اللواتي يعشن في منطقة شرق ميدلاندز بالمملكة المتحدة.

**طرق البحث:**

أجريت مقابلات جزئية الهيكلة مع نساء سعوديات يعشن حاليا في شرق ميدلاندز بالإضافة إلى استكمال استبانات دولية حول النشاط البدني. تم تسجيل المقابلات ونسخها رقميا وتحليلها باستخدام نهج موضوعي سردي؛ تم تحليل بيانات الاستبانات وصفيا. تم إجراء ما مجموعه 12 مقابلة. تم تحديد 5 موضوعات مصقولة و 19 موضوعا فرعيا.

**النتائج:**

تظهر نتائج هذا البحث أن المناخ الجذاب، وتوافر المساحات المفتوحة، وسهولة الوصول إلى الصالة الرياضية، والتكلفة المنخفضة، ونمط الحياة المستقل كطلاب، والعادات الغذائية، والنقل الفعال، وثقافة المملكة المتحدة كميسرين للنشاط البدني، وانخفاض توافر الصالات الرياضية للنساء فقط، وعدم مشاركة مدربي اللياقة البدنية، وانخفاض الاهتمام بالفئات العمرية المختلفة كعوائق للنشاط البدني في شرق ميدلاندز بين النساء السعوديات.

**الاستنتاجات:**

على الرغم من أن النتائج من النساء السعوديات، فإنه من منظور المشاركات أنه يمكن اتخاذ إجراءات أكثر ملاءمة لزيادة الوعي بمزايا النشاط بين النساء بشكل عام. بالاعتماد على نموذج تقديم الخدمة على أساس مراحل التغيير وإشراك أصحاب المصلحة المعنيين في تطوير وتنفيذ التدخلات الفعالة، يجب تطوير الاستراتيجيات والسياسات لتعزيز مشاركة النساء السعوديات والمغتربات من البلدان الأخرى في النشاط البدني بالمملكة المتحدة .

## Introduction

Physical activity plays a critical role in maintaining health and improving quality of life.^1^ Accordingly, data from several studies suggest that the health benefits of physical activity are associated with a reduction in the occurrence of many non-communicable diseases, some of which, such as cardiovascular disease, diabetes mellitus, hypertension, malignancy, obesity, and depression, are among the leading causes of vascular inflammation and atherosclerosis.^2^, ^3^, ^4^, ^5^ In addition to improving several clinical conditions, regular physical activity has a profound beneficial effect on the immune system, thereby strengthening the immune response and preventing several infectious diseases.^6^^,^^7^ One study identified the Saudi Arabian population as possessing several characteristics that increase their high risk modifiable cardiovascular factors, physical inactivity being one of them.^8^

Recent evidence from the Department for Digital, Culture, Media, and Sport (DCMS) in the UK suggests that, in 2017–18, 63.3% of the people living in Britain aged 16 and over were considered physically active. During this period, however, other ethnic groups, including Arabs, were reportedly 60.9% less physically active than the overall average—a trend that has continued steadily since 2015–16.^9^ In contrast to the above findings from cross-sectional studies, reliable data from the World Health Organization (WHO) that includes people from all age groups revealed that approximately 58.5% of the Saudi population are physically inactive, of which 52.1% are men and 67.7% are women.^10^

Exercise is defined as a subcategory of physical activity, a structured, planned, and repetitive activity aimed at improving or maintaining physical fitness.^11^ The most recent guidelines by the WHO and other national agencies highlighted that several environmental factors such as the fear of outdoor violence, increased traffic, air pollution, insufficient car parks, and a lack of sports facilities and sidewalks contribute to the increasing percentage of people with insufficient physical activity.^12^ A recent systemic review recognised increased residential development, traffic congestion, unpleasant weather, cultural obstacles, lack of social support, lack of women's physical activity school programmes, and a lack of time and resources as factors that result in lower physical activity levels among Saudi Arabian women.^13^

In KSA, the main barriers preventing Saudi female university students from taking up exercise were the lack of supportive facilities.^14^ Another study showed that lack of facilities, cultural traditions, and lack of time were considered the leading causes of physical inactivity for Saudi women.^15^ Another study confirmed that even though female students in KSA have more knowledge regarding physical activity benefits than male students, they are less physically active.^16^ It is well understood and commonly recognised that different societies have different values, which may differently affect health outcomes.^17^ Although social and demographic variables affect health-promoting activities, the findings of studies about the impacts of these factors have been inconsistent.^18^ These findings concur with the limited available literature on physical activity in Saudi Arabian female students. To add to the existing literature, this study explored the possible obstacles to and facilitators of physical activity among Saudi Arabian women living away from their country – more precisely, those living in the East Midlands region of the UK.

## Materials and Methods

### Research design

This study was based on interpretive phenomenology and sought to investigate how women perceive facilitators and barriers to physical activity, as well as to comprehend and interpret the phenomenon through shared knowledge and experiences.^19^ Semi-structured interviews were chosen for the data collection as they are the preferred method to gain extensive information, particularly about the experiences, attitudes, and beliefs of the interviewees.^20^

Furthermore, purposive selection was used to ensure that the sample reflected a range of ages and different cities in the East Midlands.

### Data collection and analysis

The recruitment email was sent to all Saudi societies in East Midlands’ universities and Saudi clubs under the KSA Cultural Bureau in the UK, inviting them to forward the information to all students and independents. The advertising poster was also distributed in all the mosques in Nottingham and all community centres. Participants were considered eligible for inclusion if they were women over 18 years of age and a Saudi Arabian national.

The consent form and participant information sheet were sent to interested and eligible participants by email to provide enough time for them to read and ask any questions before the interview. Notably, signed informed consent was obtained before conducting the interviews. The interview questions were based on a review of the relevant literature and the expert opinion of the project's supervisor. The interviews sought opinions on physical activity, current physical activity knowledge, differences in physical activity between the UK and KSA, perceived facilitators and barriers to physical activity (see appendix for the interview guide). Three pilot interview was conducted which helped modify the interview guide and ensure understanding and neutrality.^21^ The semi-structured interviews were conducted face-to-face and lasted an average of 30–45 min. Though the participants were fluent in English, to capture rich data, a few of the interviews were also conducted in Arabic. The interviews were recorded using a digital voice recorder (Sony recorder icd-px240) in a private room within the University of Nottingham. The 12 interviews were completed and no more were conducted due to data saturation had been reached.^21^ The interview transcripts in Arabic were translated into English by the author and double-checked with another professional transcriber to ensure translation validity. Before the interview, the short version of the International Physical Activity Questionnaire (IPAQ) was administered to evaluate the participants' physical activity level.^23^ Moreover, a thematic narrative approach and framework analysis was created based on the codes discovered. The researcher coded all transcripts and the supervisor observed the process to ensure consistency of discovered themes and any discrepancies discussed and resolved. Manual coding was used to organise and manage interview transcripts.^24^ The credibility of the data was ensured by having the author and the project's supervisor independently code and interpret the data, providing a basis for reflective discussions which helped provide a more complete understanding and enhance the reliability of the data. Disagreements on coding were resolved and independent coding and re-reading of transcripts were performed to ensure consistency in interview technique.^25^

## Results

### Demographic data

A total of 20 responses were received, of which 12 were eligible for inclusion in the study. Data saturation was achieved after interviewing the 12 participants. Eight interviews were conducted in English, two in Arabic, and two in mixed Arabic and English. The mean participant age was 31.8 years and the mean length of time the participants had lived in the UK was three years. The majority of participants lived in Nottingham and all of them were students (seven pursuing master's degrees and five PhDs). According to the IPAQ short version analysis guidelines, half of the participants were considered low-level physically active, five moderately physically active, and one participant was identified as highly physically active (23*).* The participant demographics are summarised in Table 1. On performing a thematic analysis, a total of five refined themes and 19 sub-themes were identified (Table 2).

**Table 1:** Participants’ demographic data.

Participants	Age	Moved to the UK	Occupation	IPAQ scoring
P1	32 years	Five years	PhD student	Low
P2	31 years	Three years	PhD student	Low
P3	32 years	Two years	Master Student	Moderate
P4	31 years	Two years	Master Student	High
P5	28 years	One year and six months	Master Student	Low
P6	27 years	Two years	Master Student	Low
P7	27 years	Four years	PhD student	Moderate
P8	30 years	Two years	Master Student	Moderate
P9	35 years	Two years	Master Student	Moderate
P10	42 years	Nine years	PhD student	Moderate
P11	33 years	Two years	PhD student	Low
P12	33 years	Two years and six months	Master Student	Low

**Table 2:** Refined themes and sub-themes.

Refined Themes	Sub-Themes
1.1 Benefits of Physical Activity	1.1.1 General improvement of health 1.1.2 Mental health 1.1.3 Physical health 1.1.4 Family benefits
1.2 Definition of Physical Activity	3.2.1 Type of activity 3.2.2 Scheduling of activity
3.3 Barriers and Facilitators of Physical Activity in the UK	3.3.1 Environmental 3.3.2 Facilities 3.3.3 Lifestyle 3.3.4 Transportation 3.3.5 Cultural 3.3.6 Barriers to Physical Activity in the UK
3.4 Barriers and Facilitators to Physical Activity in KSA	3.4.1 Facilities 3.4.2 Environmental 3.4.3 Transportation 3.4.4 Cultural 3.4.5 Facilitators
3.5 Recommendations	3.5.1 To be more physically active in KSA 3.5.2 To be more physically active in the UK

### Benefits of physical activity

#### General health improvement

Most participants (n = 11) commented about the benefits of physical activity in improving health, boosting metabolism, disease prevention, and weight loss: ‘*It can boost the metabolism, it can lower the susceptibility of future diseases like osteoporosis or osteoclasts and other bone diseases’* *(P3).* Participants also identified advantages of physical activity in terms of improving energy, blood circulation, weight loss, adrenaline hormone stimulation, and contributing to tissue growth: *‘Losing weight and stimulating the hormone adrenaline in the body helps improve digestion and helps tissue growth naturally’ (P8).* Physical activity was also considered to improve brain function and as a treat pain: *‘The more you do physical activity the more your brains work (P10)*; *‘Improves our kind of physical problems, for example, as I mentioned, my shoulder and neck pain’ (P11)*.

#### Mental health

Respondents expressed many positive comments about the mental health benefits of physical activity. These included relieving stress from studying (n = 4), providing positive energy (n = 2), and promoting happy feelings (n = 2): ‘*makes me happy’ (P2); ‘gives you a chance to get all negative energy out of the body so you can think positively’ (P6)*. The most surprising remark was that physical activity was considered a medication and helped in decreasing symptoms of depression: *‘It's helped me with my depression as well. I have taken Prozac as an antidepressant for maybe two years. When I started to exercise consistently, I found* *my symptoms improved and they decreased the dose’ (P2)*.

#### Physical health

The physical advantages mentioned include keeping the body fit, improving body shape, and maintaining a good healthy lifestyle: *‘The basic benefit of the physical activity is that it is very useful to maintain body shape and physical health’ (P12*).

#### Family benefits

Interestingly, one participant described physical activity as being beneficial to the whole family as it gives a chance to strengthen the relationship between mothers and their children and to spend more time together being physically active rather than sitting and watching television: *‘We enjoy being together and doing physical activities and that's a good point for me and for them [children] and also for the relationship between mom and children’ (P2).*

### Definition of physical activity

#### Type of activity

The participants defined physical activity as any activity like walking, running, domestic and workplace activities, or going to a gym: *‘The activity we do every day like walking or doing our work in the home or in the workplace or it could be the activity we do in the gym centre’ (P1)*. It was also seen as any movement that leads to health enhancement: *‘I believe that it is a movement that I do to enhance my health and its different components’ (P9).* It is not necessarily well-structured work, it could be any kind of movement like house cleaning or shopping: *‘Doing some activities that are out of your comfort zone, so it can include cleaning the house, moving, going shopping, coming back, or going to work’ (P10).*

#### Scheduling of activity

The physical activities are not fixed for a specific time or restricted to a specific schedule; it is a lifestyle: *‘It is done during my free time; there is no timetable’ (P1).*

### Barriers to and facilitators of physical activity in the UK

#### Environmental

The weather is a significant factor that leads to participants being more active compared to the climatic conditions in KSA: *‘If I want to exercise at 7 am in Saudi or here it is different because it is hot under the sun there compared to the UK, because in the UK the weather can help with exercise outside the home’ (P1).* Also, the availability of parks and natural environments contributes to participants being more physically active, and this increases endurance: *‘Here in the UK, the atmosphere and the natural factors are all more helpful than KSA’ (P12).*

#### Facilities

The most exciting aspect revealed by the data was the accessibility of facilities in the UK. The availability and feasibility of the gyms are considered significant factors for activity in the UK and the numerous types of activity (i.e. indoor and outdoor) that are possible: *‘I think here it is easier to do exercise than in Saudi’ (P1); ‘Here there are lots of gyms’ (P3); ‘The gym is open here 24 hours’ (P9)*. The personal trainers are also a factor that facilitate more physical activity in the UK: ‘*I've got a personal trainer, and that helps me a lot in planning my training’ (P6).*

Moreover, the gym costs are cheaper in the UK than in KSA, which may increase the chance for individuals to be more physically active. The gyms are well-equipped and offer a variety of classes for all age groups. In fact, they also provide special student prices: *‘Me and my two children, two boys, monthly we pay around 60 pounds and use the facilities for gym any time’ (P2); ‘they've offered – the gym – to give the students a discount to join the gym’ (P8).*

Furthermore, the gym employees helped participants be more active by providing clear explanations and encouragement, thereby making it more interesting and professional for them in the gym: *‘I go to the gym, and they explain to us every single thing, and they try to encourage me. They also do in Saudi, but I think here they encourage the people to come to the gym more than in Saudi’ (P1).*

Also, more attention is paid to all age groups in the family – including children and the elderly – being a part of any activity. It is also safer in the UK to take part in physical activity: *‘In the UK we care about children as they can do swimming, horseback riding, we do not have those activities in KSA’ (P8)*; *‘safety is higher than in KSA and freedom is more so there is a lot of potential for walking’ (P12).*

#### Lifestyle

The lack of social life and student life contribute to having more accessible and organised time for the children and their physical activities: *‘I found myself here to be more organised and I have more time for my children because there are no social activities with family’ (P2).*

Regular student life and independent lifestyles are considered a factor in being more active: *‘Well maybe because I'm more independent, I have to do many things by myself. Like I'd have to go to the supermarket by myself’ (P4).*

Dietary lifestyle is also a major positive factor facilitating better heath: ‘*Because in the UK I'm eating more healthy food as it's affordable, I can buy berries, I can buy yoghurt or anything I want, and it's really cheap or cheaper than KSA’ (P3)*; or a negative factor (to be more unhealthy): ‘*So I eat lots of sugar, and I don't like cooking, the first thing I eat in the morning is some cake and biscuits, cookies, and then I eat something from restaurants like fast food junk food’ (P6).* Moreover, lifestyles differ between the UK and KSA: *‘the lifestyle here is different in the UK because you familiarise yourself with going for walks’ (P7); ‘Because the people here in the UK are used to doing these things, to go to the gym every day, walking to work or the university, unlike in Saudi’ (P7).*

#### Transportation

One factor which relates to being physically active is the transportation system. In the UK, there is no need for a vehicle to reach any destination; this encourages individuals to walk there or to reach it by bus or train: ‘*The transportation is also a factor as you can walk here whenever you want, but in Saudi, I think it is not possible*’ *(P3);* ‘*In the UK I walk a lot, I go from my home to the university by walking, and I do everything by walking, I do not have a car and I do not know how to drive*’ *(P4).*

#### Cultural

Most participants believed that the UK culture was one of the major causes for more physically active (n = 9). As such, they can wear their hijab without any trouble while being physically active and engaging in activities like swimming: *‘You know without any inconvenience issue, they can you know wear their hijab and do their exercise and they do not find, you know, difficulties for me at all unlike before’ (P2).* The overall rules are more flexible; there is greater individual freedom and less social commitments in the UK: ‘*I think there is more freedom to do stuff because I am not worried about people around me’ (P4); ‘The social life here in the UK is that we are more flexible, we do not have a lot of people who gather together for a social commitment’ (P5); ‘In the UK they do outdoor sport, I do any sport because the culture and social status lag behind here’ (P5).* People around you can also encourage physical activity: *‘People in the UK, my friends, my Chinese friends, my friends from all over the world they're encouraging me to do so’ (P4)*; and the family to take part in group workouts: ‘*In the UK you can do it with your husband which you cannot do in KSA because the gyms are separated’ (P9).*

#### Barriers to physical activity in the UK

Despite participants identifying several major factors for being more physically active in the UK, some obstacles may lower physical activity in the UK. These barriers include combined gyms or limited accessibility of separate gyms for women, and academic and family commitments. Separate fitness centres for women may also have old and non-specialised equipment: *‘A lot of gyms, but there is only one that is suitable for me as wearing a hijab I can't go to mixed gyms. And lack of professional equipment’ (P4); ‘Doing yoga and kinds of mixed classes with men and women and I did not find it comfortable at all. Maybe I was feeling guilty about it, maybe it is not the right thing to do to exercise with men’ (P11).*

### Barriers and facilitators to physical activity in KSA

#### Facilities

Interestingly, there were variations in both beneficial and adverse reactions to physical activity in KSA. There was complete consensus in terms of gym costs being a barrier to physical activity, also in addition to limited availability of gym centres: *‘It is too expensive compared to here. For example, she paid for a year eight thousand riyals for the gym and here it is you know, I think we pay 30 pounds per month or less than 30 pounds’ (P1).* Low accessibility of personal trainers and less experience discourage Saudi Arabians from becoming more physically active: *‘When I was in KSA I never went to a gym for many reasons. The first one when I was trying to find a personal trainer. It didn't convince me to have a personal trainer’ (P3); ‘But never asked me what my goals are and what they would offer me’ (P11).* Furthermore, lack of knowledge and fewer facilities are another reason people in KSA are less active: *‘The lack of awareness between Saudi and UK. The UK has more awareness about physical activity, how it's important to do this exercise more than KSA’ (P8).* Moreover, the poor availability of excellent gyms and separate gyms for women hinders them from being more active: *‘The gyms in KSA are impressive, but the professionalism is questionable; in comparison, some sports machines in the UK are old, and there are no women's gyms’ (P11).* Swimming facilities for women in KSA are not clean or simple to utilise: ‘*You can do every kind of swimming skills you want to do in the UK but there in KSA, they are small, or they're not clean’ (P10).*

#### Environmental

The climate in KSA is one of the biggest obstacles to physical activity: *‘But whether you consider that number one, you know KSA always has hot weather or cold weather’ (P2).* The absence of encouragement has also resulted in less activity: *‘Also there is no motivation required in KSA, which supports the lack of going to the gym compared to the UK where stimulation and encouragement exist’ (P8).*

#### Transportation

Contributing factors to being less physically active are the distance to the gym, the lack of public transport, and the availability of cars: *‘I live in Mecca in KSA so we don't have that many gyms there, I have to drive for more than half an hour to go to the gym’ (P3) ‘The obstacles of transportation, I cannot drive and I was an employee, and at the same time I studied, and I could not continue in the gym’ (P12).*

#### Cultural

The lack of cultural and parental awareness had an impact on the level of physical activity: *‘People in KSA used to not be aware of the importance of the gym, for example, my parents didn't see a benefit of going to the gym if you're not fat’ (P4).* Social lifestyle, social habits, and social beliefs are also some of the most critical factors reducing physical activity: *‘Because, for example, when we gather with our relatives, we decide on a place where we go and sit the whole time. We bring snacks and food. We don't move. If you want to meet, we go to restaurants. So we don't have active social activities like now in the UK. I can go walking with a group of people’ (P4).* However, awareness regarding physical activity has increased in recent years in KSA: ‘*But I think nowadays there's a massive change even in Saudi towards activity – moving physical activity’ (P7).*

#### Facilitators

Most of the respondents reported Islam as being one of the factors encouraging them to be more active: *‘I don't think it is anything to do with religion because it is all about the people and how they want to live their life’ (P3); ‘Wearing a hijab will not affect the practise of* *physical activity’ (P5).*

### Recommendations

#### To be more physically active in KSA

The participants provided many recommendations to increase physical activity levels in KSA, such as increasing the availability of gyms, reducing the price of gyms, respecting values and beliefs, raising awareness of the importance of physical activity through social media, and increasing the accessibility and requirements of facilities: *‘Social media. It is important to educate people about sports and now many use it, and it attracts people’ (P7); ‘I think we in KSA, we need more awareness about the importance of physical activities for women’ (P11).*

#### To be more physically active in the UK

Many recommendations were made by respondents for Saudi women living in the UK to be more engaged in physical activity. These suggestions included opportunities of less expensive gym facilities, excellent weather that contributes to walking more, and practising physical activity as a group or team with friends. It was argued that this was the right time to be more physically active as they have less social and cultural obligations and responsibilities: ‘*I think it's a good opportunity to be more active here and enjoy the extent of the lifestyle’ (P3); ‘In the UK it's a chance for someone to start a healthy lifestyle’ (P4)*.

## Discussion

From the participants' overall response, the current study found that they have good knowledge about the benefits of physical activity; nevertheless, half the participants exhibited low levels of physically activity. Also, environmental factors, facilities, lifestyle, culture, gym accessibility, and ease of transportation were identified as factors that contribute to Saudi women being physically active in the UK. Conversely, reduced access to high-quality physical activity facilities, a lack of knowledge, social responsibilities, unsuitable weather, high gym membership prices, and cultural and parental constraints were identified as barriers to Saudi women being more physically active in KSA.

There is evidence to support the findings that Saudi Arabian female students in the United States and the KSA have similar knowledge of the benefits of physical activity.^16^ Most respondents reported an improvement in study-induced stress levels with exercise; correspondingly, all the participants agreed about the clear benefits of physical activity. More notably, one participant succeeded in improving their symptoms of depression and reduced their depression medication after engaging in regular exercise.

Most recent data from the UK's DCMS indicates that Asian ethnic groups were less physically active in 2017–18 compared to the general average—a trend that has remained stable since 2015–16.^9^ This difference was also observed in the current study participants' levels of physical activity. As per the IPAQ responses collected as part of this study, more than 50% of the participants fall within the low physical activity category. These results are inconsistent with those of an Arab adolescent lifestyle study in KSA performed on a large group of adolescents from three major cities. The results of a questionnaire survey showed that physical activity levels among adolescent males were somewhat moderate (average prevalence was 55.5%) and very low among females (average prevalence was 21.9%).^26^

Well-structured research observed that children of active mothers were twice as likely as children of inactive mothers to be active.^27^ Other evidence showed that family physical activity aggregation occurred in 73% of the study group between mothers and their children.^28^ In the current study, two participants state that their mother-and-children relationship grew more substantial after engaging in physical activity in the UK, offering health benefits to the entire family, making everyone active, and reducing sedentary activities like sitting and watching television for prolonged periods.

Environmental factors, general facilities, lifestyle, culture, accessibility of gyms, and ease of transportation are all factors that facilitate Saudi women being physically active in the UK. Two other studies recognise environmental factors such as sidewalks, street connectivity, population density, and land-use diversity as factors that support greater physical activity.^29^^,^^30^ However, some environmental factors, such as traditional perceptions among Saudi Arabian women and others of physical activity being embarrassing or intimidating, act as barriers.^29^^,^^30^

The non-availability of facilities emerged as another key barrier. A US-based study shows that it is hard for young women to engage in physical activity because of restricted access to facilities.^31^ The importance of the accessibility of equipment as a significant environmental factor affecting physical activity seems to be increasing.^32^ Moreover, combined-sex gyms, reduced availability of separate gyms, and the absence of new sports equipment were considered obstacles to physical activity in the UK. In light of the evidence from the Asian population in the UK, providing more attention to issues of modesty as well as gender segregation and security would enhance the accessibility of recreational physical activity in this target group.^33^

Another study acknowledged that the British-Bangladeshi population of Muslim women reported that the sportswear was not suitable. It could attract attention to their body, and their cultural convictions in relation to music and pictures were inconsistent with gyms.^34^^,^^35^ Contrastingly, other evidence indicates that Islamic culture-related problems such as dress code are not seen as obstacles to physical activity in Saudi Arabian women.^14^ The participants in this study agree that Islamic culture is not a barrier to physical activity.

In this study, reduced access to high-quality physical activity facilities, lack of information, social obligations, unsuitable climate, high gym membership prices, lack of efficient transport systems, reduced personal vehicle availability, cultural and parental constraints, and social practices were factors that Saudi women perceive as obstacles to physical activity in KSA. This finding, which is supported by Samara et al., shows that the primary obstacles preventing Saudi female university students from exercising are the absence of support facilities.^14^ Research shows that the absence of facilities, cultural traditions, time constraints, and stress were deemed the major causes of Saudi women's inactivity.^15^^,^^36^^,^^37^ The latest systemic review from KSA found that physical activity could be most influenced by volatile modifications in climate, traffic congestion, cultural barriers, absence of social support and programmes for physical activity in female college students as well as the absence of time and resources.^13^ The participants made certain recommendations to help women increase physical activity levels in KSA, such as increasing gym availability, lowering gym prices, raising awareness of the significance of physical activity through social media, and improving accessibility and equipment availability.

On the other hand, the participants suggested that factors encouraging Saudi Arabian women to be physically active in the UK were opportunities for less expensive gyms, excellent weather, and fewer social and cultural obligations. Physical activity counselling support and routine physical activity evaluation and promotion in clinical practice can help reduce healthcare usage and expenses.^4^^,^^38^ This is one of the most important recommendations and should also be implemented in KSA, thereby enabling the Saudi Arabian population to gain multiple physical activity benefits.

In addition to following a qualitative approach to explore the factors influencing physical activity levels in Saudi Arabian women living in the UK, the current study obtained physical activity data from the participants using the IPAQ. This significantly increased the reliability and validity of the results obtained and informed the discussion of the results and their significance. The authors have not had any professional or personal contact with any of the study participants, thereby ensuring there was no researcher bias. However, a severe limitation to the study relates to the generalisability of the results to the wider context of Saudi Arabian women living away from home. Specifically, this research was carried out among Saudi women living in the East Midlands region of England. The opportunities, facilities, and barriers available to Saudi women elsewhere may differ substantially from that available in the East Midlands, and hence the results of this study would not be applicable in such circumstances.

## Conclusions

This study has provided an account of the barriers and facilitators to physical activity among Saudi Arabian women living in the East Midlands in the UK. Evidence from this study indicates that most of the participants were familiar with the advantages of physical activity. Despite their knowledge, half of them exhibited low levels of physically activity. Access to walking and bike riding areas and gyms and their incorporation into the planning process by the health authority should be a priority in KSA to promote the concept of health through the participation in physical activity.

## Recommendations

Channels such as social media should also be utilised to promote physical activity and raise awareness in KSA. Moreover, further studies are required to investigate the magnitude of these obstacles and facilitators to physical activity involving a large sample of Saudi Arabian women in the UK.

## Source of funding

This research did not receive any specific grant from funding agencies in the public, commercial, or not-for-profit sectors.

## Conflict of interest

The author declares no conflict of interest.

## Ethical approval

The study was approved by the University of Nottingham Research Ethics Committee (Reference 230-1901) (30-April-2019).
