# Supplementary material for: Perceived barriers and facilitators of physical activity among Saudi Arabian females living in the East Midlands
Source: J Taibah Univ Med Sci. 2021 Dec 14;17(3):384–91. doi: 10.1016/j.jtumed.2021.11.002 (PMC9170784; doi:10.1016/j.jtumed.2021.11.002)
Supplement: Multimedia component 1 [file mmc1.docx]

**Appendix: Interview Guide**

In the beginning, there is an introduction to the interview and then an explanation of the purpose of the interview.

| **Questions** | **Potential prompts** |
| --- | --- |
| Tell me briefly, what is the physical activity? | What is the physical activity? |
| What are some of the key things that have helped or facilitated you to get involved in physical activity? | What is the factor that helps you to be more active? |
| Tell me about your experience in accessing your local sporting or physical activity facilities in the UK and Saudi Arabia. |  |
| What have been some of the challenges or barriers to you participating in physical activity in the UK and Saudi Arabia? | - What influence does cost or money have on how active you are? - What influence does transport have on how active you are? - What influence does the weather have on how active you are? |
| What are the differences in physical activity between the UK and Saudi Arabia? |  |
| What key recommendations would you make for Saudi Arabian women living in the UK and Saudi Arabia to be more physically active? |  |
